# Behavioral Phenotyping in a Murine Model of *GBA1*-Associated Parkinson Disease

**DOI:** 10.3390/ijms22136826

**Published:** 2021-06-25

**Authors:** Jenny Do, Gani Perez, Bahafta Berhe, Nahid Tayebi, Ellen Sidransky

**Affiliations:** Medical Genetics Branch, National Human Genome Research Institute, National Institutes of Health, Bethesda, MD 20892, USA; do.jennyt@gmail.com (J.D.); gani.perez@nih.gov (G.P.); bahafta.berlhe@nih.gov (B.B.)

**Keywords:** Gaucher disease, *GBA1*, Parkinson disease, mouse model, buried pellet test, novel object recognition

## Abstract

Mutations in *GBA1*, the gene encoding glucocerebrosidase, are common genetic risk factors for Parkinson disease (PD). While the mechanism underlying this relationship is unclear, patients with *GBA1*-associated PD often have an earlier onset and faster progression than idiopathic PD. Previously, we modeled *GBA1*-associated PD by crossing *gba* haploinsufficient mice with mice overexpressing a human mutant α-synuclein transgene (*SNCA^A53T^*), observing an earlier demise, shorter life span and faster symptom progression, although behavioral testing was not performed. To assess whether *gba^+^*^/−^//*SNCA^A53T^* mice exhibit a prodromal behavioral phenotype, we studied three cardinal PD features: olfactory discrimination, memory dysfunction, and motor function. The longitudinal performance of *gba^+^*^/*−*^//*SNCA^A53T^* (*n* = 8), *SNCA^A53T^* (*n* = 9), *gba^+^*^/*−*^ (*n* = 10) and wildtype (*n* = 6) mice was evaluated between ages 8 and 23 months using the buried pellet test, novel object recognition test and the beam walk. Fifteen-month-old *gba^+^*^/*−*^//*SNCA^A53T^* mice showed more olfactory and motor deficits than wildtype mice. However, differences between *gba^+^*^/*−*^//*SNCA^A53T^* and *SNCA^A53T^* mice generally did not reach statistical significance, possibly due to small sample sizes. Furthermore, while *gba* haploinsufficiency leads to a more rapid demise, this might not result in an earlier prodromal stage, and other factors, including aging, oxidative stress and epigenetics, may contribute to the more fulminant disease course.

## 1. Introduction

Parkinson disease (PD) is a late-onset neurodegenerative disorder with a complex etiology, afflicting 2% of the population over age 65 worldwide [1,2]. It is primarily characterized by bradykinesia and tremor, and the pathological finding of Lewy bodies in the substantia nigra and brainstem. Lewy bodies are the abnormal conglomeration of multiple proteins, including α-synuclein and ubiquitin, although their exact role in PD pathogenesis remains unclear [3,4,5]. Evidence suggests that dysfunctional α-synuclein found in Lewy bodies may play an influential role in PD pathogenesis, as mutations in *SNCA,* the gene encoding α-synuclein, have been implicated in familial PD [6]. In past years, the PD field has attempted to explore the relationship between α-synuclein and a common genetic risk factor associated with PD: variants in the gene *GBA1* [7].

Biallelic pathologic variants in *GBA1* are associated with Gaucher disease (GD), a recessive lysosomal storage disorder characterized by a deficiency in the hydrolyzing lysosomal enzyme glucocerebrosidase (GCase) [8]. Impaired or deficient GCase leads to its inability to cleave its native lipid substrates, glucosylceramide (GluCer) and glucosylsphingosine (GluSph), into recyclable moieties. This results in their toxic accumulation in the lysosome, leading to a wide range of symptoms [9].

Remarkably, both *GBA1* homozygotes and heterozygotes were found to have an increased incidence of PD and other Lewy body disorders. An international, multicenter study in 2009 found that patients with PD were over five times more likely to carry a pathogenic *GBA1* variant than matched controls [10]. Studies of cohorts of with dementia with Lewy bodies (DLB) and Parkinson disease dementia (PDD) have similarly consistently found an increased frequency of *GBA1* mutations, with an odds ratio of over 8 [11]. Furthermore, even patients with idiopathic PD were found to have lowered GCase levels in post-mortem samples, suggesting a correlation between GCase deficiency and PD [12]. However, the basis for this reciprocal relationship remains unclear. Furthermore, the reduced PD penetrance among *GBA1* carriers, as well as the phenotypic variation among mutation carriers with PD, complicate the issue [13].

Research investigating the biological link between *GBA1* mutations and PD has focused on how GCase dysfunction may promote or enhance the development of synucleiopathies. While both loss-of- and gain-of-function hypotheses have been posited, it has also been shown that, in vitro, GCase and α-synuclein directly interact in the lysosome, and that raising GCase activity in mice and iPSC cells results in reduced α-synuclein levels [14,15,16]. These studies highlight the potential for GCase augmentation as a therapy for not only *GBA1*-associated PD, but idiopathic PD as well. While treatments have been developed for patients with GD, including recombinant enzyme replacement therapy and substrate reduction therapy, they do not cross the blood–brain barrier. Since drugs improving neural GCase activity might also reduce α-synuclein levels and thereby improve PD prognosis, studies are underway to develop therapies that can overcome the blood-brain barrier and rescue neuronal pathology [17].

Animal models have often contributed to our understanding of disease pathogenesis and have facilitated drug development. Various attempts to model *GBA1*-associated PD in the mouse have included chemical models, genetic models and a mixture of both, as recently reviewed [18]. Genetic mouse models to elucidate the biological link between *GBA1* and PD vary widely in mutation type and severity. Most GD murine models do not exhibit parkinsonian features unless combined with a second challenge, such as α-synuclein overexpression or prosaposin deficiency [19]. We and others have cross-bred *gba* haploinsufficient mice with mice carrying a mutated human α-synuclein transgene (*SNCA^A53T^*) to exacerbate symptom progression and shorten survival time relative to *SNCA*^A53T^ mice [20,21,22].

Comprehensive characterizations of these *GBA1*-associated PD mouse models have included neurochemical, immunostaining and, at times, behavioral evaluations [15,23]. Since different studies lack consistency in both the type of testing and the age at which it is performed, comparisons between different *GBA1*-associated PD mouse models are difficult. Additionally, since PD is an age-related neurodegenerative disorder, mice may only experience an observable decline in behavior later in life [24].

This study characterizes mice at different ages to evaluate memory, olfaction, and motor coordination as three prodromal behaviors in mice with *gba* haploinsufficiency and A53T α-synuclein overexpression [20]. These haploinsufficient mice with an *SNCA*^A53T^ transgene (*gba^+^*^/*−*^//*SNCA^A53T^*) have an earlier disease onset and a more rapid course than mice with *SNCA^A^*^53T^ alone, but the age when phenotypic differences began was unclear. In this study, mice underwent longitudinal behavioral evaluations assessing three cardinal behavioral phenotypes seen in patients with PD: impairments in cognition, olfaction and motor coordination. Our aims were to identify behavioral changes that may precede the more overt, rapid decline in health of these mice, and to discern whether such prodromal signs were exacerbated by *gba* haploinsufficiency. While we found indications that behavioral deficits developed prior to overt symptomology in *gba^+^*^/*−*^//*SNCA^A53T^* mice, we did not find significant evidence suggesting that these mice had an earlier or more severe prodromal stage relative to *SNCA^A53T^* mice.

## 2. Results

### 2.1. Novel Object Recognition (NOR)

The novel object recognition test assesses the memory of a mouse by measuring the amount of time the mouse spends near a novel object versus a familiar object. Mice with a functional memory will generally display a natural curiosity toward new objects by interacting more frequently with them, while those with memory problems will interact less frequently with novel objects. The results are quantified via a preference score, which is the percentage of time spent interacting with the novel object versus the total time spent interacting with either the novel or familiar object.

**Figure 1:**
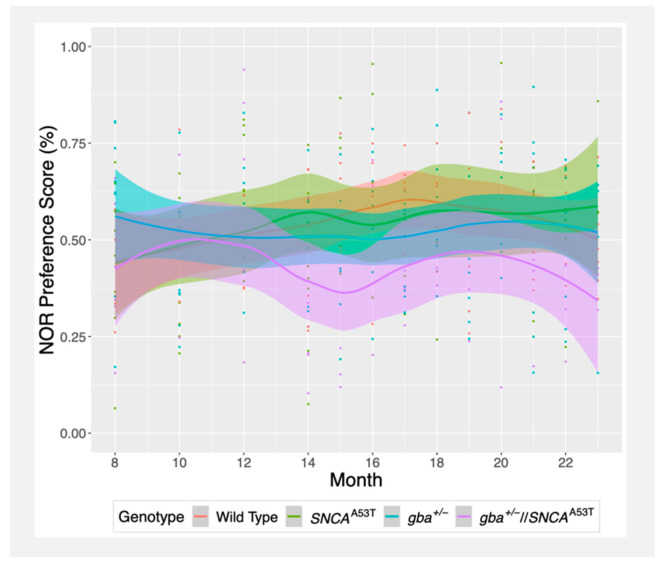
Evolution of novel object recognition (NOR) preference scores over time, grouped by genotype. Trend lines were calculated via LOESS regression. Colored filled regions represent standard error. Points represent measurements for each mouse, with the different colors indicating genotype.

Both *gba^+^*^/*−*^ and wildtype mice exhibited preference scores that stayed near 50% over the testing window, indicating a random interaction between the two objects (Figure 1). Surprisingly, *SNCA^A53T^* mice showed preference scores near 50% at 8 months, with scores that improved over time, though not significantly relative to wildtype mice. In contrast, *gba^+^*^/*−*^//*SNCA^A53T^* mice demonstrated a novel preference score just under 50%, which decreased to around 40% after 15 months of age. This drop in preference scores of *gba^+^*^/*−*^//*SNCA^A53T^* mice after 15 months resulted in a lower overall slope compared to *gba^+^*^/*−*^ and *SNCA^A53T^* mice, though the difference was not significant (Figure 1, Table 1). Overall, these results suggest that *gba^+^*^/*−*^//*SNCA^A53T^* mice may develop memory deficiencies after 15 months, which are exacerbated relative to the other groups, though larger sample sizes may be needed to properly resolve whether the differences persist between these groups.

NOR preference scores did not correlate with beam walk latency, suggesting that motor dysfunction did not affect performance on the memory assay. Similarly, NOR results were not significantly associated with the time taken to find the buried pellet in the olfactory test (Figure S1A).

### 2.2. Buried Pellet Test

**Figure 2:**
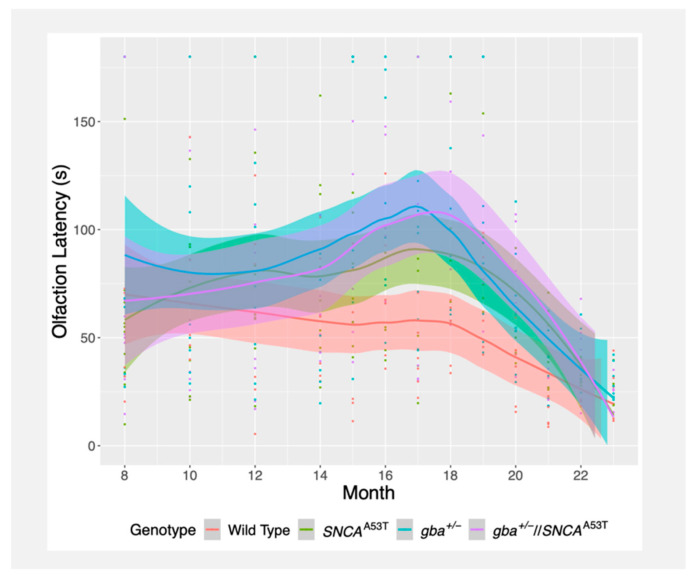
Olfaction latency over the 8–23-month testing window, measured based on the buried pellet test. Trend lines were calculated via LOESS regression. Colored fill represents standard error; colored points represent measurements, coded by genotype.

The buried pellet test measures the overall olfactory ability of mice by measuring the amount of time that a mouse takes to uncover a sweet-smelling buried treat from underneath a layer of bedding. Compared to wildtype mice, *gba^+^*^/*−*^//*SNCA^A53T^*, *gba^+^*^/*−*^, and *SNCA^A53T^* mice all exhibited increased latency to locate the buried pellet at age 8 months, though not statistically significant. Similarly, *gba^+^*^/*−*^//*SNCA^A53T^*, *gba^+^*^/*−*^, and *SNCA^A53T^* mice all showed reduced improvement over time relative to wildtype mice, though this too did not reach significance (Figure 2, Table 1).

Curiously, each group of mice showed an overall improvement in time taken to find the buried pellet over the 8–23-month testing window, which was largely driven by a marked upswing in performance across groups after month 19. This improvement was potentially a learning effect due to the repeated testing paradigm. Prior to month 20, *gba^+^*^/*−*^//*SNCA^A53T^*, *gba^+^*^/*−*^, and *SNCA^A53T^* mice all showed an increase in olfaction latency relative to wildtype mice, with the slope of the *gba^+^*^/*−*^//*SNCA^A53T^* mice reaching statistical significance (*p* = 0.028) (Table S1). However, there was no meaningful difference in baseline or latency change over time between *gba^+^*^/*−*^//*SNCA^A53T^* and *SNCA^A53T^* mice, indicating that gba haploinsufficiency did not significantly exacerbate olfactory symptoms (Table 1). Overall, these results suggest that *SNCA^A53T^* mice gradually develop olfactory deficits prior to the presentation of overt PD-like symptoms, though these symptoms are not significantly enhanced by *gba* haploinsufficiency. 

Latency to locate a pellet did not correlate strongly with performance on the beam walk, indicating that motor dysfunction did not significantly impede the animal’s ability to locate and extract food (Figure S1B).

### 2.3. Traverse Beam Walk

**Figure 3:**
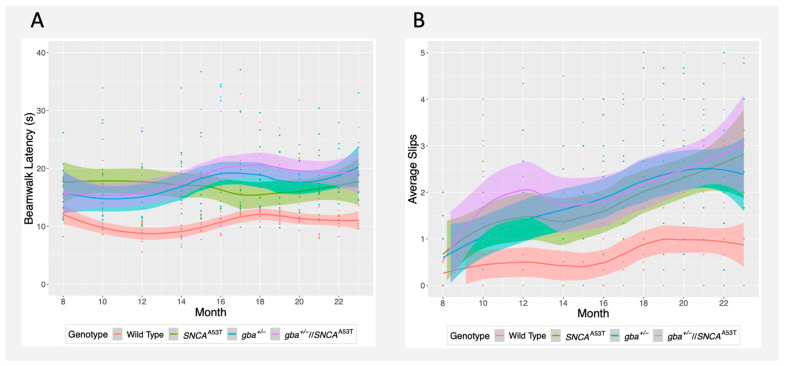
Beam walk latency and slip frequency over time. (**A**) Beam walk latency over time. Trend lines were calculated via LOESS regression. Colored fill represents standard error. Colored points represent measurements, coded by genotype. (**B**) Evolution of slip frequency over time, recorded between ages 8 and 23 months.

The traverse beam walk assesses motor ability of the mice by observing their ability to walk across a thin, 48 inch-long beam. Motor function was assessed by measuring both the time taken to traverse the beam and the number of misplaced steps (slips) observed while crossing. *SNCA^A53T^*, *gba^+^*^/*−*^, and *gba^+^*^/*−*^//*SNCA^A53T^* mice were significantly slower at crossing the beam than wildtype mice after age 8 months (*p* = 0.005, 0.017, and 0.014, respectively), although there was no significant difference in the change of beam walk latency over time. This suggests that impaired motor performance may have developed prior to the 8–23-month testing window (Figure 3A, Table 1). On the other hand, while all groups rarely slipped off the beam at 8 months, the change in slip frequency over time was statistically significant for *SNCA^A53T^*, *gba^+^*^/*−*^, and *gba^+^*^/*−*^//*SNCA^A53T^* mice when compared to wildtype mice (*p* = 0.0047, 0.0165, 0.0142) (Figure 3B, Table 1). However, there was no significant difference in slips or beam walk latency between the three groups, suggesting that *gba^+^*^/*−*^//*SNCA^A53T^* mice do not exhibit exacerbated motor symptoms relative to the *gba^+^*^/*−*^ or *SNCA^A53T^* mice.

As expected, there was a significant correlation between slip frequency and time taken to cross the beam (*p* = 0) (Figure S1C).

### 2.4. Hindlimb Clasp

**Figure 4:**
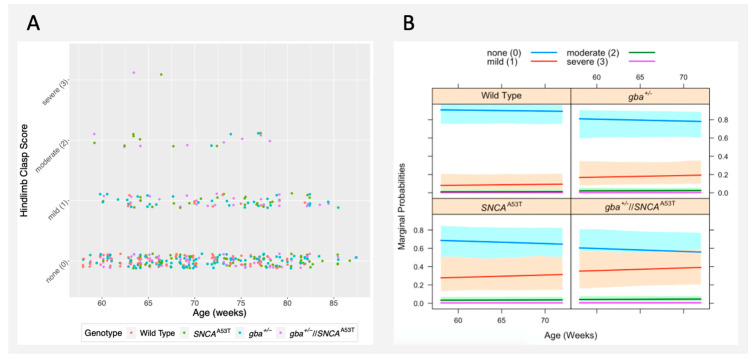
Hindlimb clasp performance: (**A**) hindlimb clasp observations recorded during the testing window (55–90 weeks), colored by genotype; (**B**) model-predicted probability of displaying a given phenotype during a particular week. Colored lines display the chance of exhibiting scores of 0 (no phenotype), 1 (mild phenotype), 2 (moderate phenotype), and 3 (severe phenotype). Graphs are segregated by genotype. Colored fill represents standard error.

The hindlimb clasp (HLC) test is used as a marker of neurodegeneration in some mouse models, including those with certain cerebellar ataxias. Hindlimb clasp is assessed on an ordinal scale (0–3), with higher values indicating a more severe phenotype. In general, most mice within our cohorts showed no (0) phenotype or a mild (1) phenotype, with very few mice showing a moderate (2) or severe (3) phenotype. Of the 21 observations rated as moderate or severe, 18 (86%) were in *SNCA^A53T^* or *gba^+^*^/*−*^//*SNCA^A53T^* mice (Figure 4A).

Both *SNCA^A53T^* mice and *gba^+^*^/*−*^//*SNCA^A53T^* mice exhibited an increased HLC score when compared to wildtype mice (*p* = 0.027, 0.009; odds ratio = 4.39, 6.17), with mice showing approximately a 30% and 40% chance (respectively) to exhibit at least a mild phenotype by age 60 weeks (Figure 4B). In contrast, wildtype mice exhibited an approximately 10% chance of exhibiting a mild phenotype by 60 weeks, which remained level over the testing period. *gba^+^*^/*−*^ mice showed a slightly higher incidence of an abnormal phenotype relative to wildtype mice, though this did not reach significance (Figure 4B, Table 2). 

## 3. Discussion

This study characterized the age-related behavior and motor function in *gba^+^***^/^***^−^***//***SNCA^A53T^* mice compared to their controls (*gba^+^***^/^***^−^*, *SNCA^A53T^*, and wt), with the goal of evaluating whether earlier disease manifestations were apparent in *gba^+^***^/^***^−^***//***SNCA^A53T^* mice, analogous to the prodromal signs observed in patients with *GBA1*-associated PD. Discerning PD early in the disease course could aid in identifying disease biomarkers, and in understanding the disease pathogenesis. Furthermore, such findings could serve as endpoints for evaluating therapies for PD. We did observe significant differences in olfaction, slip frequency over time, and hindlimb clasp between *gba^+^***^/^***^−^***//***SNCA^A53T^* and wildtype mice. We also found that *gba^+^***^/^***^−^*//*SNCA^A53T^* mice tended to have more challenges with the beam walk latency and memory performance test than wildtype mice, although the values were not statistically significant.

Various studies have shown that patients with PD carrying *GBA1* mutations have an earlier average age of onset and a more rapid disease course relative to those with idiopathic PD [25,26,27]. Similarly, *gba^+^***^/^***^−^***//***SNCA^A53T^* mice have an earlier age of onset and a faster time to death compared to *SNCA^A53T^* mice [20]. However, it was unclear whether *GBA1* mutations in PD conferred a more severe deterioration of prodromal features relative to non *GBA1*-associated PD in mice or humans. In this study, there were no significant differences between the early performance of *gba^+^***^/^***^−^***//***SNCA^A53T^* mice and *SNCA^A53T^* mice, suggesting that *gba* haploinsufficiency may not exacerbate prodromal symptoms in these mice. Some clinical studies have similarly found no difference when comparing motor and nonmotor symptoms of *GBA1*-associated PD cases versus idiopathic PD [28], although the literature is conflicted in this regard [29].

However, it is plausible that the testing frequency incompletely captured the behavioral decline of *gba^+^***^/^***^−^***//***SNCA*^A53T^ mice, which may have progressed with more rapidity in the weeks or days prior to overt symptomology. Notably, overt symptomology progresses much faster in *gba* haploinsufficient *SNCA^A53T^* PD mice, where symptoms develop within a few hours prior to death, while *SNCA^A53T^* mice may survive up to a week after presentation of overt symptoms [20]. If *gba^+^***^/^***^−^***//***SNCA^A53T^* mice similarly experience a more rapid and severe decline in behavioral symptoms within the weeks prior to death, a monthly testing frequency may have been insufficient to fully reveal the extent of the behavioral decline. 

It was indeed surprising that *gba*^+/−^ mice appeared to have diminished performance on the beam walk and olfaction tests, despite never developing PD-like pathologies such as α-synuclein aggregation or dopaminergic neuron depletion [20]. This may suggest that *gba* haploinsufficiency has some effect on these modalities, independent of *SNCA^A53T^* overexpression. More recent studies have found support for this hypothesis, identifying subtle reductions in olfaction and motor coordination in *GBA1* mutation carriers and GD homozygotes relative to healthy controls, even without progression to clinically diagnosed PD [30,31]. 

The behavioral pattern observed with the *gba^+^***^/^***^−^***//***SNCA^A53T^* mice is consistent with other mouse models of *GBA1*-associated PD, such as the *gba*^L444P/L444P^ and *gba*^D409V/wt^ point mutation models. *gba*^D409V/wt^ mice exhibit an age-dependent cognitive decline at 12 months of age on the Y-maze and Morris Water Maze, two assays designed to assess memory and learning [24]. While investigators tracked behavior from 3 to 12 months of age, behavior was not followed after one year. Mice with *SNCA^A53T^* have previously been shown to perform poorly on the NOR and buried pellet test compared to their wildtype controls [32].

Other parameters that may contribute to the behavioral decline observed are anxiogenic and dysphoria-like measures. Anxiety and depression are common comorbidities associated with PD in patients, and some PD mouse models similarly exhibit evidence of anxiety and depression-like behavior on the open field test and forced swim test [33,34]. 

Ultimately, the data presented here suggest that early signs of parkinsonism can be observed in *gba^+^***^/^***^−^***//***SNCA^A53T^* mice, as reflected by their hindlimb clasp and less favorable performance in memory, olfactory, and motor assays. However, this performance was not statistically different than that observed in *SNCA^A53T^* mice, indicating that *gba* haploinsufficiency, while leading to an earlier demise, may not have an earlier or more severe prodromal phase. One major shortcoming of this study was the limited sample size, which may have made it difficult to obtain statistical significance with small differences. Furthermore, in this study, the age at symptom onset in *SNCA^A53T^* mice was quite variable, and thus comparisons at uniform timepoints may not reflect the same stages in disease progression. In retrospect, the study may have benefited from the performance of secondary assessments of olfaction and memory. 

The lack of significant differences in prodromal behavior between *SNCA^A53T^* mice with and without a null *gba* allele may indicate that the enhanced symptom severity observed in this model may not have had an earlier start. This suggests that a combination of *gba* haploinsufficiency and other factors, such as aging, oxidative stress, and epigenetics, may contribute to the onset and disease duration in this mouse model and merits further evaluation. Regardless, this study provides a crucial longitudinal assessment of this mouse model’s motor and nonmotor phenotype, and suggests that olfaction may be a useful endpoint when evaluating therapies in *gba^+^***^/^***^−^***//***SNCA^A53T^* mice.

## 4. Materials and Methods

### 4.1. Mice

*Gba1*-knockout mice (B6;129S6-*gba^tm1Nsb^*/J, JAX Stock No: 002594) and α-synuclein transgenic mice (B6;C3-Tg(Prnp-SNCA*A53T)83Vle/J, JAX Stock No: 004479) were crossed with a C57BL/6 background (JAX Stock No#: 000664) as previously described [20]. *gba^+^*^/*−*^//*SNCA^A53T^* lines were generated by crossing *gba^+^*^/*−*^ mice with *gba^+^*^/*+*^//*SNCA^A53T^* mice, and male and female mice littermates were studied. All procedures were approved by the National Human Genome Research Institute’s Animal Care and Use Committee. In this study, four groups of mice were followed, including 6 *wt*/*wt*, *9 gba^+^*^/*−*^, 10 *gba+*/*+*//*SNCA^A53T^*, and 8 *gba^+^*^/*−*^//*SNCA^A53T^*. 

### 4.2. Testing Schedule

The behavioral battery was comprised of three tests, run on separate days during the course of one week. Mice were tested once every other month between ages 8 and 14 months. As they approached the terminal endpoint age described in [20], testing increased in frequency to monthly until 22 months of age.

Mice were tested first with the novel object recognition assay, next with the buried pellet test, and lastly with the beam walk assay. Mass and hind limb clasp scores were recorded monthly.

### 4.3. Novel Object Recognition (NOR)

The NOR assay was performed as described [35]. Briefly, mice were habituated in an 18 × 18 × 11 inch plastic arena with two identical (“familiar”) objects (glass bottles and small blocks) placed on either side of the arena. Objects were selected to be heavy enough to resist movement and tall enough to prevent the mouse from climbing on top. Mice were permitted to interact with the arena for 5 min before being returned to their home cage. Then, 24 h later, mice were reintroduced to the cage where one of the objects was exchanged with a novel object of similar size but with different features. Novel object location was randomized using a random number generator. The amount of time spent interacting with the novel and familiar object during a five-minute interval was measured using a camcorder recording from above and analyzed using TopScan 3.0. Mice were excluded from analyses if they exhibited freezing behavior for 60 consecutive seconds, interacted with either object for less than 2.5 s, or interacted with either object for more than 120 s. Preference score was calculated as follows: Score (%) = (Time_Novel_)/(Time_Novel_ + Time_Familiar_). Different objects were utilized for each NOR assay. 

### 4.4. Buried Pellet Test

Prior to testing, mice were habituated to the artificially sweetened flavored dry cereal Froot Loops in their home cage. Olfaction tests were conducted after an 18 h fast [36,37,38]. Weight loss never exceeded 10% of the mouse’s weight (Figure S2). After their fast, mice were placed in a new cage with 1 inch of fresh bedding. Froot Loops were buried approximately 0.5 cm under the bedding at one of ten random spots in the cage, selected using a random number generator. Mice were placed in the center of the cage and the amount of time required to locate the buried cereal was recorded. If the mouse interacted with the Froot Loop but did not eat it, it was assumed that it was not motivated to search for food and the data were excluded from that month’s trial. After the trial, mice received food ad libitum once again. Water was provided freely during the entire food restriction period.

### 4.5. Traverse Beam Walk

Mice were placed on one end of an elevated, rounded narrow beam 0.75 inches in diameter, 8 inches in height, and 48 inches in length, and were exposed to bright, aversive light at the starting point. As the mouse traversed the beam from the exposed start point to the darker finish point, motor coordination was assessed by measuring the number of slips (defined as foot placement below the lower half of the beam) and the time required to traverse the beam [39]. Mice were tested three times per month, and the time taken to traverse the beam and slip frequencies were averaged.

### 4.6. Hindlimb Clasp Score (HLC Score)

The HLC score was evaluated weekly, starting from age 14 months. HLC is an indicator of cerebellar ataxia and precedes the symptomatic onset of hindlimb paralysis [40]. Briefly, mice were suspended over their home cage by their tails for 10 s and assessed using the following scale: 0 = limbs are consistently splayed outwards for 10 s; 1 = one hind limb is retracted towards the abdomen, or is touching the abdomen for 5 s; 2 = both hind limbs are retracted towards the abdomen, or are touching the abdomen for 5 s; and 3 = both hind limbs exhibit consistent clasping, or are touching the abdomen, for 10 s.

### 4.7. Statistical Analysis

For beam walk and olfactory tests, between-group effects were analyzed with longitudinal linear mixed models using the package *lme4* (Version 1.1.23) in R (Version 4.0.2). Novel object recognition preference scores were analyzed with a beta-distributed, generalized linear model using the package *glmmTMB* (Version 1.0.2.1). In each model, genotype (*gba^+^*^/*−*^//*SNCA^A53T^*, *SNCA^A53T^*, *gba^+^*^/*−*^, and wildtype), time (8–23 months), and the genotype–time interaction were included as fixed effects, and slopes and intercepts were allowed to vary by mouse ID. Models were fit using the maximum likelihood estimator.

Hindlimb clasp data were analyzed with a backwards ordinal continuous ratio model using the package *GLMMadaptive* (Version 0.8) in R. As suggested, in the ordinal mixed-model vignette, hindlimb clasp scores were transformed into cohorts HLC ≤ 1 and HLC ≤ 2 and fitted to the model HLC ~ cohort + genotype + age using a binomial mixed-model regression. Intercepts were allowed to vary by mouse ID.

Correlation between measured parameters were also analyzed with mixed-effects models using the packages referenced above. In each analysis, one parameter was used as the dependent variable and the other was included as a fixed effect, with slopes and intercepts allowed to vary by mouse ID.

## Figures and Tables

**Table 1:** Results of the longitudinal mixed-effects models for the three behavioral parameters assessed. *p*-values comparing both the intercept (9-month baseline score) and slope values between genotypes are provided. Significant *p*-values (*p* < 0.05) are underlined and in bold. For all tests except the novel object recognition test, a more positive slope value indicates worsening performance over time.

Test	Genotype	Baseline Value (8 Months)	Slope	*p*-Val vs. Wildtype (Baseline)	*p*-Val vs. Wildtype (Slope)	*p*-Val vs. *gba^+^*^/*−*^ (Baseline)	*p*-Val vs. *gba^+^*^/*−*^ (Slope)	*p*-Val vs. *SNCA^A53T^* (Baseline)	*p*-Val vs. *SNCA^A53T^* (Slope)
Novel Object Recognition	Wildtype	0.503 ± 0.051 ^1^	0.0049 ± 0.0052 ^1^	~	~	~	~	~	~
	*gba^+^* ^/*−*^	0.520 ± 0.041 ^1^	0.0006 ± 0.0044 ^1^	0.790	0.532	~	~	~	~
	*SNCA^A53T^*	0.473 ± 0.043 ^1^	0.0086 ± 0.0052 ^1^	0.666	0.461	~	~	~	~
	*gba^+^*^/*−*^//*SNCA^A53T^*	0.464 ± 0.043 ^1^	−0.0043 ± 0.0051 ^1^	0.580	0.225	0.884	0.493	0.985	0.055
Buried Pellet Test	Wildtype	76.1 ± 11.3	−3.07 ± 1.18	~	~	~	~	~	~
	*gba^+^* ^/*−*^	105.0 ± 9.2	−3.36 ± 0.97	0.057	0.849	~	~	~	~
	*SNCA^A53T^*	80.6 ± 9.2	−1.24 ± 1.06	0.758	0.249	~	~	~	~
	*gba^+^*^/*−*^//*SNCA^A53T^*	88.9 ± 10.0	−1.54 ± 1.10	0.401	0.344	0.071	0.214	0.545	0.841
Beam Walk Latency	Wildtype	10.0 ± 1.45	0.08 ± 0.13	~	~	~	~	~	~
	*gba^+^* ^/*−*^	15.4 ± 1.81	0.27 ± 0.11	**0.028**	0.549	~	~	~	~
	*SNCA^A53T^*	18.5 ± 1.45	−0.03 ± 0.13	**0.007**	0.260	~	~	~	~
	*gba^+^*^/*−*^//*SNCA^A53T^*	16.3 ± 1.60	0.27 ± 0.13	**0.014**	0.314	0.693	0.969	0.299	0.105
Beam Walk Slips	Wildtype	0.28 ± 0.46	0.05 ± 0.04	~	~	~	~	~	~
	*gba^+^* ^/*−*^	0.91 ± 0.37	0.17 ± 0.03	0.294	**0.017**	~	~	~	~
	*SNCA^A53T^*	1.31 ± 0.37	0.21 ± 0.04	0.089	**0.005**	~	~	~	~
	*gba^+^*^/*−*^//*SNCA^A53T^*	1.31 ± 0.40	0.19 ± 0.04	0.101	**0.014**	0.684	0.796	0.816	0.690

^1^ Coefficients are derived from logistic-transformed linear regression.

**Table 2:** Results of the backwards ordinal mixed-model regression of hindlimb clasp scores. Odds ratio values indicate the chance that a given mouse genotype will have an abnormal HLC score relative to wildtype mice. Values in parentheses represents the 95% confidence interval. *p*-values comparing incidence of abnormal HLC score between genotypes are also provided. Significant *p*-values (*p* < 0.05) are underlined and in bold.

Test	Genotype	Odds Ratio Relative to Wildtype	*p*-Val vs. Wildtype	*p*-Val vs. *gba^+^*^/*−*^	*p*-Val vs. *SNCA^A53T^*
Hindlimb Clasp	Wildtype	~	~	~	~
	*gba^+^* ^/*−*^	2.29 ^1^ (0.65–8.09)	0.196	~	~
	*SNCA^A53T^*	4.39 ^1^ (1.19–16.23)	**0.027**	~	~
	*gba^+^*^/*−*^//*SNCA^A53T^*	6.17 ^1^ (1.57–24.24)	**0.009**	0.098	0.571

^1^ Coefficients are derived from logistic-transformed linear regression.

## Data Availability

All supporting data is available upon request.

## Supplementary Materials

The following are available online at https://www.mdpi.com/article/10.3390/ijms22136826/s1.
