# Supplementary material for: Behavioral Phenotyping in a Murine Model of GBA1-Associated Parkinson Disease
Source: Int J Mol Sci. 2021 Jun 25;22(13):6826. doi: 10.3390/ijms22136826 (PMC8267726; doi:10.3390/ijms22136826)
Supplement: Supplementary file 1 [file ijms-22-06826-s001.zip › ijms-1257308-supplementary.pdf]

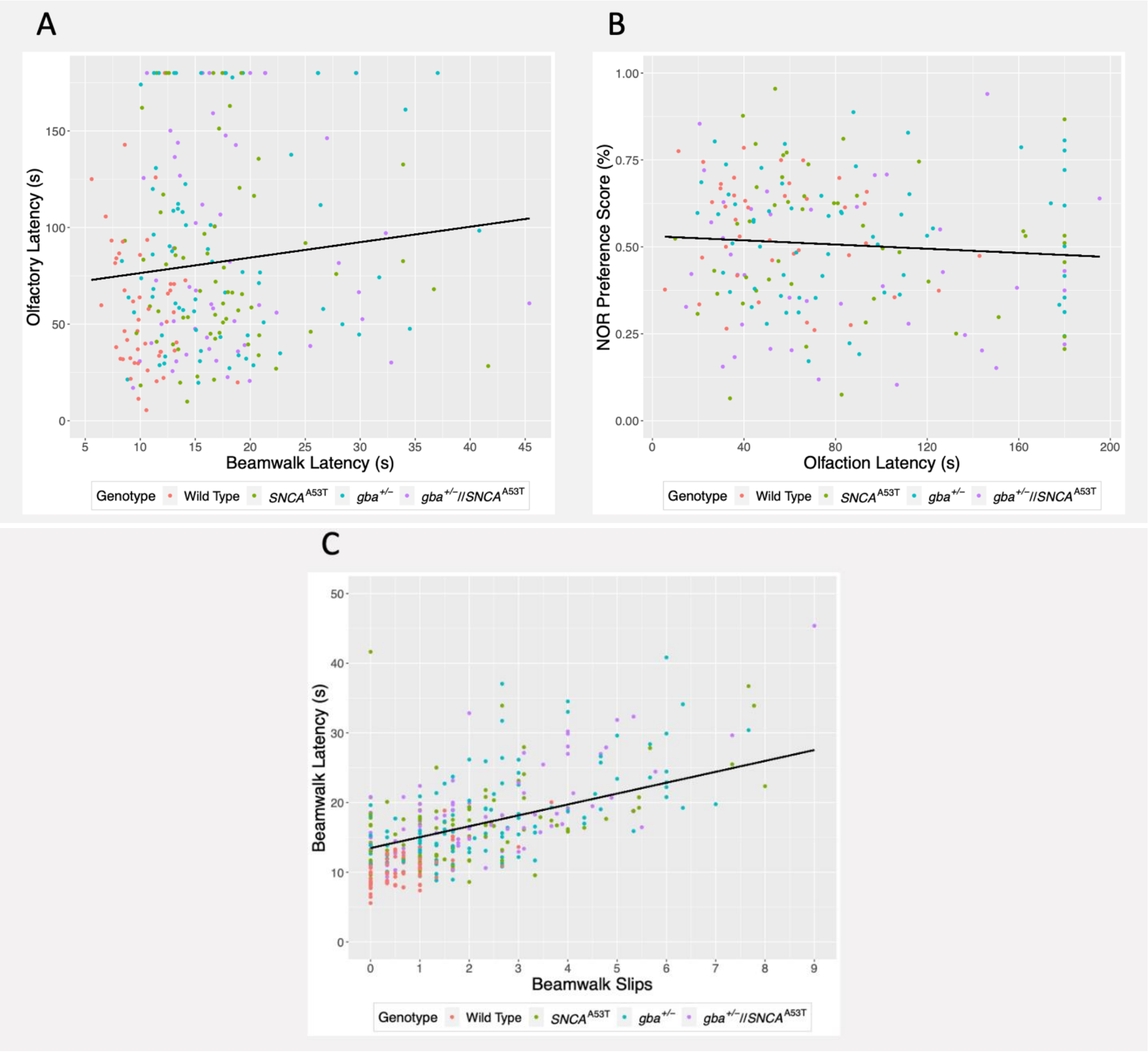

Figure S1. Correlation analyses between testing modalities; (**A**) correlation analysis between preference scores and olfaction latency as determined by the buried pellet test. The black line displays the best-fit line after controlling for repeated measures Figure 0. (**B**) Correlation analysis between olfaction latency and time taken to cross the beam walk. There was not a significant correlation between these parameters (p = 0.099); (**C**) Correlation between beam walk latency and slip frequency from the beam walk test. The correlation between these parameters was statistically significant (p = 0).

Table S1. Results of linear mixed model of olfaction data with observations after month 19 removed. P-values comparing both the intercept (9-month baseline score) and slope values between genotypes are provided. For all tests except the novel object recognition test, a more positive slope value indicates worsening performance over time.

Test	Genotype	Baseline Value (8 months)	Slope	p-val vs. Wild Type (Baseline)	p-val vs. Wild Type (Slope)	p-val vs. *gba*$^{+/-}$ (Baseline)	p-val vs. *gba*$^{+/-}$ (Slope)	p-val vs. *SNCA*^{A53T} (Baseline)	p-val vs. *SNCA*^{A53T} (Slope)
Buried Pellet	Wild Type	66.5±13.4	-0.98±1.85	~	~	~	~	~	~
	gba$^{+/-}$	73.9±11.0	2.04±1.52	0.444	0.209	~	~	~	~
	SNCA^{A53T}	56.3±10.7	3.31±1.63	0.808	0.084	~	~	~	~
	gba$^{+/-}$*//SNCA*^{A53T}	56.3±11.8	4.69±1.76	0.815	**0.028**	0.283	0.257	0.999	0.567

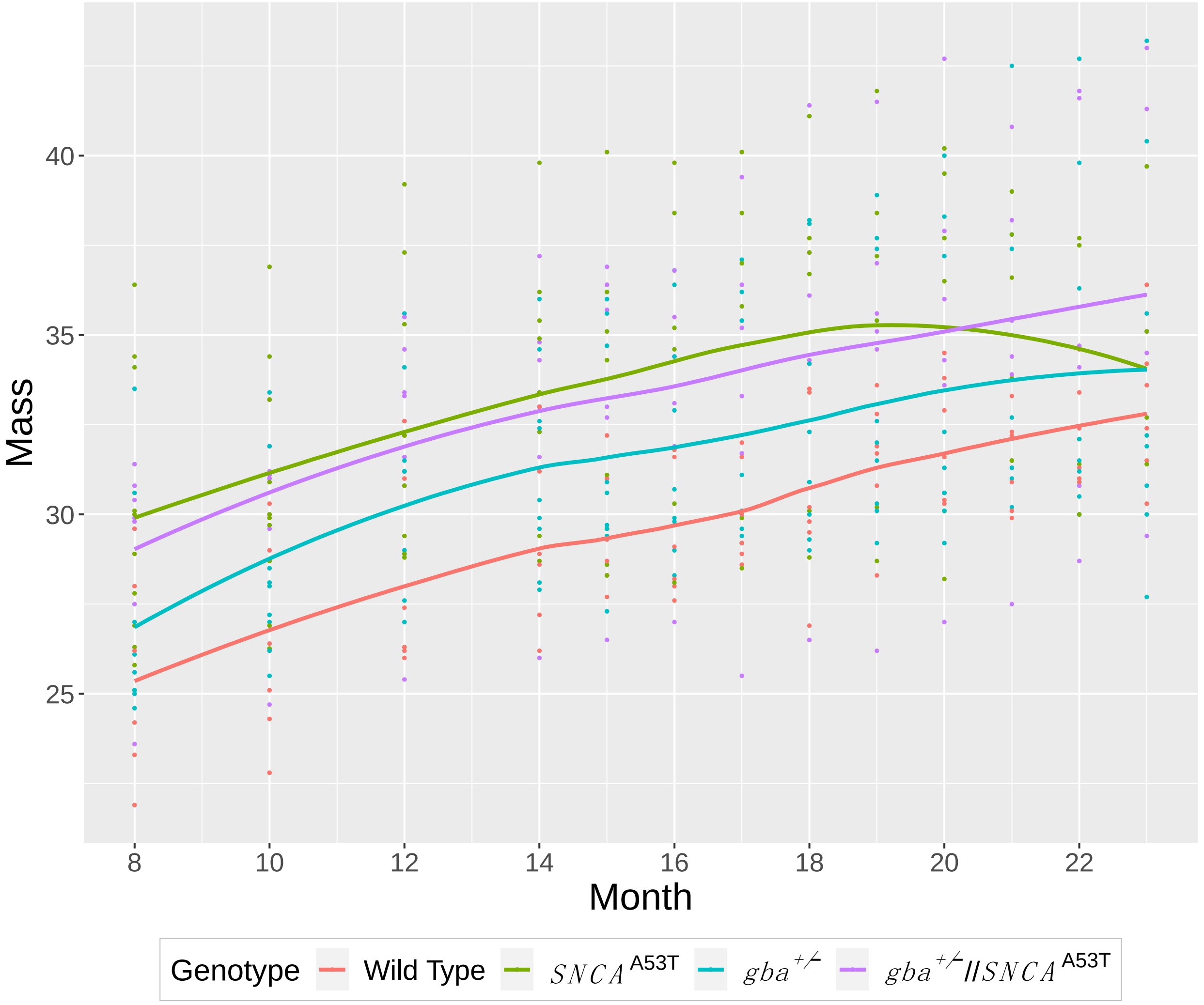

Figure S2. Mouse mass over time. Trend lines were calculated via LOESS regression. Colored fill represents standard error. Colored points represent individual observations, coded by genotype.
